# In Vitro Study of a Superhydrophilic Thin Film Nitinol Endograft that is Electrostatically Endothelialized in the Catheter Prior to the Endovascular Procedure

**DOI:** 10.3390/jfb7040031

**Published:** 2016-11-29

**Authors:** Mahdis Shayan, Yanfei Chen, Puneeth Shridhar, Colin P. Kealey, YoungJae Chun

**Affiliations:** 1 Department of Industrial Engineering, University of Pittsburgh, Pittsburgh, PA 15213, USA; mahdis.shayan2@gmail.com (M.S.); yanfeichen@pitt.edu (Y.C.); 2 Department of Bioengineering, University of Pittsburgh, Pittsburgh, PA 15213, USA; pus8@pitt.edu; 3 Business Development, Neurosigma, Inc., Los Angeles, CA 90024, USA; ckealey@neurosigma.com; 4 McGowan Institute for Regenerative Medicine, Pittsburgh, PA 15219, USA

**Keywords:** thin film nitinol, electrostatic cell seeding, biocompatibility, endovascular devices

## Abstract

Electrostatic endothelial cell seeding has evolved as an exceptional technique to improve the efficiency of cell seeding in terms of frequency of attached cells and the amount of cell adhesion for the treatment of vascular diseases. In the recent times, both untreated and superhydrophilic thin film nitinol (TFN) have exhibited strong prospects as substrates for creation of small-diameter endovascular grafts due to their hallmark properties of superelasticity, ultra low-profile character, and grown hemocompatible oxide layer with the presence of a uniform endothelial layer on the surface. The purpose of the current study is to understand the effects of endothelial cell seeding parameters (i.e., applied voltage, incubation time, substrate chemistry, and cell suspension solution) to investigate the cell seeding phenomenon and to improve the cell adhesion and growth on the TFN surface under electrostatic transplantation. Both parallel plate and cylindrical capacitor models were used along with the Taguchi Design of Experiment (DOE) methods to design in vitro test parameters. A novel in vitro system for a cylindrical capacitor model was created using a micro flow pump, micro incubation system, and silicone tubings. The augmented endothelialization on thin film nitinol was developed to determine the effect of cell seeding and deployed in a 6 Fr intravascular catheter setup. Cell viability along with morphology and proliferation of adhered cells were evaluated using fluorescent and scanning electron microscopy. Our results demonstrated that the maximum number of cells attached on STFN in the catheter was observed in 5 V with the 2 h exposure of in the cell culture medium (CCM) solution. The condition showed 5 V voltage with 0.68 × 10^−6^ µC electrostatic charge and 5.11 V·mm^−1^ electric field. Our findings have first demonstrated that the electrostatic endothelialization on the superhydrophilic thin film nitinol endograft within the catheter prior to the endovascular procedure could enhance the biocompatibility for low-profile endovascular applications.

## 1. Introduction

Vascular diseases are known as the leading cause of morbidity and mortality worldwide. The majority of vascular diseases are found in small-caliber blood vessels such as smaller than 6 mm in luminal diameter [1,2,3]. One of the examples is bypass surgery, which is the typical treatment used to restore blood flow in occluded small blood vessels by use of one or more healthy blood conduits. While both arterial and venous allogenic grafts have been used as substitutes for small vascular grafts, the inflammatory reaction and calcification, as well as the limited availability are the major restricting factors for allografts [4]. Occurrence of thrombosis and restenosis result in the failure of vascular grafts in short term such that nearly 25%–40% of initially successful of these procedures fail within one year [5].

To address these issues, synthetic materials including expanded polytetrafluoroethylene (e-PTFE) and Polyethylene terephthalate (Dacron^©^) polyester were recently tested and used to develop vascular grafts in order to overcome the problems. E-PTFE is an inert polymer with an electronegative charged luminal surface (similar to endothelium layer in the native blood vessel) that is antithrombotic and demonstrated successful results for lower limb bypass grafts (7–9 mm). Dacron^©^ polyester has demonstrated an excellent result in large-diameter vascular grafts in thoracic and aortic regions. However, both e-PTFE and Dacron^©^ polyester showed poor patency rates if used for small-caliber vascular grafts [4]. In a randomized trial to compare the e-PTFE with saphenous vein for femoro-popliteal bypass, the patency rate for e-PTFE and saphenous vein are reported as 37% and 70% after 54 months, respectively even though both of these showed 92% patency after 1.5 months [4]. Implanted e-PTFE and Dacron^©^ grafts do not develop a well-grown confluent endothelialized lumen spontaneously, leading to adhesion of platelets and development of a luminal fibrin layer that can lead to thrombosis. In addition to thrombosis, other causes of vessel narrowing, such as intimal hyperplasia expedite the occlusion. As a result, restenosis results in critical narrowing in 30%–50% of patients after these synthetic vascular grafts implantation [5]. Recent new studies showed the biocompatibility enhancement for e-PTFE and Dacron^©^ polyester grafts by covering a layer of endothelial cells in a similar structure of the native blood vessel’s luminal surface in order to minimize or to prevent any potential thrombogenic or renarrowing issues [6,7,8].

Herring et al. first reported the endothelial cell seeding on the 6 mm Dacron^©^ grafts [9]. The endothelial cell seeding enhanced the patency rate after four weeks more than three times (76% vs. 22%). However, multiple limitations exist in endothelial cell seeding such as poor retention of the cells on the surface of biomaterials under blood flow shear stress, low degree of adhesion of cells, limitation of available number of cells, and time duration for harvesting and incubating cells to grow. Therefore, surface modifications using RGD (*Arginyl-Glycyl-Aspartic acid)* peptides, matrix proteins (e.g., fibronectin), growth factors, or a combination of them were used to enhance the adhesion of endothelial cells. While these strategies showed the improvement of endothelial cell adhesion, there are concerns about increasing the thrombogenicity of the surface [10,11]. In the recent years, electrostatic endothelial cell seeding (EECS) has gained attention as an alternative technique to improve the efficiency of cell seeding in terms of number of attached cells and the amount of cell adhesion. In this method, a positive electrostatic charge is temporarily induced on the surface of the graft to promote endothelial cell adhesion [12]. Bowlin et al. not only suggested using electrostatic force to seed the endothelial cells inside the small diameter e-PTFE tubes, but also showed acceleration of endothelial cell maturation and cell retention after implant enhancement [13,14,15]. They developed a cylindrical capacitor system consisting of an internal conductor within the graft for seeding the endothelial cells on the e-PTFE vascular graft via electrostatic force. The external conductor was comprised of a stainless steel cylinder and was placed around the graft such that the internal electrode was placed at the center. Temporary positive electrical charges were induced on the e-PTFE surface as the positive electrically charged electrode and the temporarily negatively charged endothelial cells by the applied voltage were attracted to the surface of e-PTFE. It was found that the optimal electrostatic transplantation conditions are +1 V voltage and 16 min leading to seeding efficiencies of up to 90% [14]. These results are encouraging and point to significant time savings, however, the fact that e-PTFE is a non-conducting material led to difficulties for developing an apparatus due to the non-uniform contact between the metallic electrode and non-conducting scaffold materials. If one could use a conducting scaffold covered with a uniform dielectric layer, the production of a positive surface electrostatic charge is achieved by simply connecting it to a positively charged electric terminal. In addition, typical synthetic polymer grafts are too bulky to be used in small vascular applications, such as neurovascular and coronary artery bypass applications.

Our group has recently developed a novel endovascular graft that is covered by an ultra low-profile superhydrophilic thin film nitinol (STFN). While the superhydrophilic thin film nitinol has demonstrated an excellent hemocompatiblity both in vitro and in vivo, there were still concerns about the long-term biocompatibility of this new material. Therefore, we applied the electrostatic endothelialization method on our STFN graft membrane, which contains a grown oxide layer (insulator) on a conductive thin film nitinol layer. The oxide layer works as an insulator that generates positive surface charges in a similar way to the synthetic polymer graft. The endothelialization is intended to conduct prior to the endovascular procedure. The aim of the present study is to demonstrate that a superhydrophilic thin film nitinol (STFN) endograft electrostatically endothelialized in the catheter prior to the procedure represents an endovascular device that is non-thrombogenic and is ideally suited for treating various small vascular diseases.

## 2. Materials and Methods

### 2.1. Electric Field Analysis of the Parallel Plate Capacitor Model

**Figure 1:**
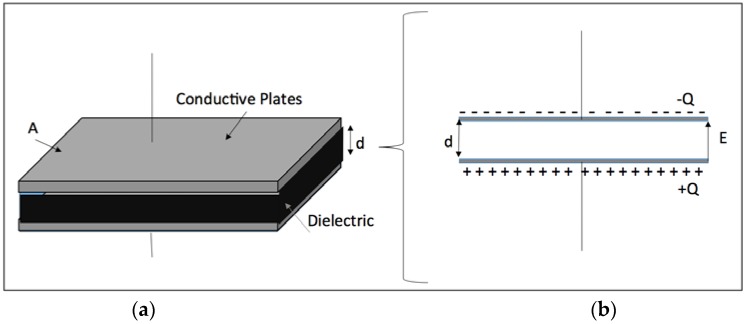
The schematic diagram of the induced electric charge (**a**) and electrical field in a parallel capacitor model (**b**).

For the parallel plate capacitor, the induced electric charge (*Q*) on the electrode surface and the induced electric field (*E*) (Figure 1) were calculated using the following Equations:

(1) Q=C×V

(2) $$E=\frac{V}{d}$$

where *V* is the applied voltage, *d* is the distance between two electrodes and *C* is the total capacitance defined by:

(3) $$C=\frac{k\varepsilon_{0}A}{d}$$

where *ε*_0_ is the permittivity constant (8.85 × 10^−12^ F·m^−1^), *A* is the surface area of electrodes and *k* is the dielectric constant which is around 80 for the cell culture medium [16]. Because no current was recorded during the experiment, the cell culture medium was assumed as dielectric material. We also ignored the capacitance of both the native and newly grown oxide layers due to the minimal thickness of these layers (i.e., nanometer scale) compared with 2 mm distance between two electrodes. The induced electric charge and electric field in each experiment were calculated based on the above Equations.

### 2.2. Electric Field Analysis of the Cylindrical Capacitor Model

**Figure 2:**
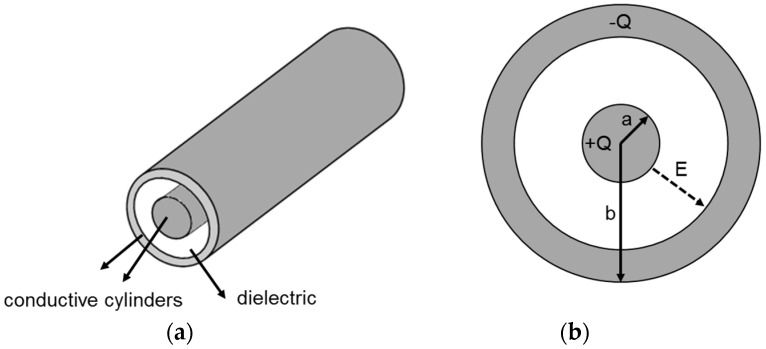
The schematic diagram of the electrostatic cell seeding with thin film nitinol with the endovascular device delivery catheter. (**a**) 3D view of the catheter with the electrode; (**b**) Cross sectional view of the cylindrical capacitor model.

For practical applications, the thin film nitinol (TFN) will be placed in the delivery catheter, which has 4–6 Fr catheter sizes (i.e., inner diameter of 1.3–2 mm). Figure 2 shows a schematic of the deployed TFN for electrostatic cell seeding inside the catheter before device delivery.

For the cylindrical capacitors composed of an inner cylinder with radius *a* enclosed by an outer cylinder with radius *b* (*b* > *a*), Gauss’ law was used to evaluate the induced electric field (*E*) and electric charge (*Q*) between the electrodes in cylindrical capacitors:

(4) $$\int EdA=\frac{\sum Q}{\in_{0}}$$

(5) $$E=\frac{Q}{2\pi rL\varepsilon_{0}}$$

(6) $$V=\overset{b}{\underset{a}{\int }}Edr=\frac{Q}{2\pi \varepsilon_{0}}\;ln\frac{b}{a}$$

(7) $$C=\frac{2\pi k\varepsilon_{0}L}{\text{ln}\;b/a}$$

where *A* is the cylindrical surface with length *L* and diameter *r*, *V* is the electrical potential and *C* is the capacitance. In our settings, radii *a* and *b* were measured as 0.19 mm and 5 mm, respectively and cylindrical surface length L is 8 mm.

### 2.3. In Vitro Cell Seeding Setup

**Figure 3:**
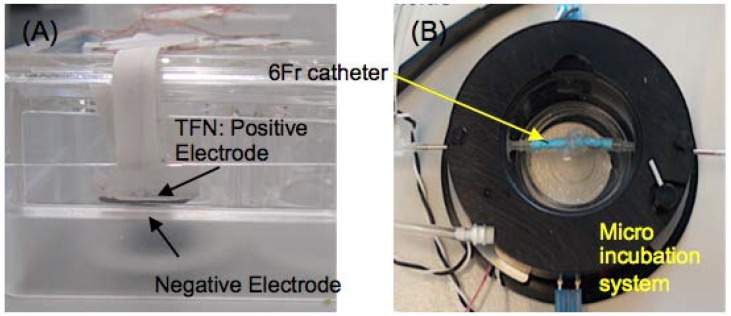
The experimental setup for (**A**) parallel capacitor model and (**B**) cylindrical catheter based model.

Both untreated TFN (UTFN) and surface-treated TFN (STFN) have been used for the experiment. Briefly, TFN was created by a DC sputter deposition [17]. While the sputtered TFN contains ~10 nm thick native oxide layer, the thicker insulating layer is important for the electrostatic cell seeding in order to generate surface charged on the insulating layer. Therefore, superhydrophilic surface treatment has been subsequently performed only for the STFN generating approximately 100 nm thick titanium oxide layer on TFN with the Hydrogen Peroxide based chemical surface treatment process [18,19]. Both UTFN and STFN were used as a positive electrode. As can be seen in Figure 3A the 3 mm × 3 mm size TFN was suspended in the cell culture medium working as a positive electrode (cathode). The TFN was placed in the cell culture medium with the distance of 2 mm from the bottom negative electrode (anode) in a 24-well tissue culture plate. A similar setup was applied to the cylindrical shape cell seeding experiment. The TFN was deployed inside the endovascular device delivery catheter (here, 6 Fr) with the negative electrode, then submerged in the micro incubation system (DH-40iL, Warner Instruments, Hamden, CT, USA) as shown in Figure 3B. Both catheter ends were connected to the circulation loop of the cell culture medium. Both temperature and CO_2_ levels were precisely controlled with the Heater Controller (TC-324C, Warner Instruments, CT) and CO_2_ Gas/pH Controller (70-2116, Harvard Apparatus, Holliston, MA, USA). Electric fields were applied using a Laboratory DC Power Supply (GPS-4303, Tecpel Co., Ltd, Taipei, Taiwan).

**Figure 4:**
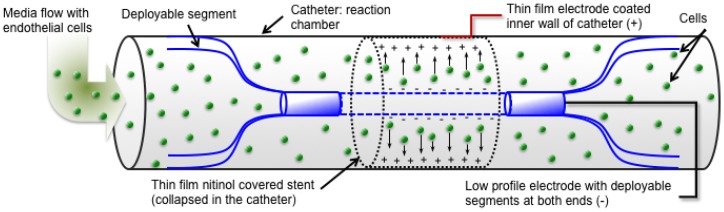
A schematic of the cylindrical shape cell seeding experimental setup.

Figure 4 represents the schematic for more details of the cylindrical shape electrostatic cell seeding experimental setup in order to show the mechanism. A TFN sample was deployed in the middle of the 6 Fr size catheter, then, a fine nitinol wire (0.014″ diameter, Nitinol.com, Fremont, CA, USA) was placed in the middle of the tube working as a negative electrode (anode). The nitinol wire has two deployable segments which help in positioning this wire accurately in the middle of the tube in order to maintain the same distance between the TFN and wire. A cell culture media that contains endothelial cells flowed into the catheter and subsequently electric field was applied to the system. The catheter was placed in a micro incubation system in order to maintain the cell culture medium at 37 °C and 5% CO_2_.

### 2.4. Design of Experiement (DOE)

To simultaneously evaluate multiple factors which can affect the level of cell seeding and behavior of seeded cells, the Taguchi method was used to design the experiments. The parameters that can potentially affect this EECS process include: the cell suspension solution, substrate chemistry, applied voltage, and incubation time. The voltage value determines both induced electric field and electrostatic charge. The range of values of these parameters is given in Table 1. For both voltage and time parameters, four levels were selected and for each suspension solution and substrate chemistry parameter, two levels were considered.

In addition to the above mentioned experiments conducted in the parallel capacitor-like set-up, experiments in the cylindrical capacitor-like intravascular catheter set-up, which have been explained above, were performed in 1 V, 5 V, and 15 V in cell culture medium. These experiments were done on STFN substrate for 30 min duration and analyzed right after the experiment (i.e., no incubation time). Analysis of variance (ANOVA) technique was utilized to identify the significant factors that affect the number of attached cells.

### 2.5. Testing the Morphology, Attachment, and Viability of Seeded Endothelial Cells

Bovine Aortic Endothelial Cell (Lonza, Allendale, NJ, USA) were grown in the medium consisting of Endothelial Cell Basal Medium (EBM)-2 (Lonza, Allendale, NJ, USA) and EGM-2 Single Quot Kit Suppl. & Growth Factors including (Hydrocortisone 0.02%, FBS 2%, VEGF 0.05%, hFGF-B2%, IGF-1 0.05% and HEGF 0.05%, GA-1000 0.05%) (Lonza, Allendale, NJ, USA). These cells at passage number 9 at a density of 2 × 10^5^ cells/cm^2^ were suspended in the cell culture medium (CCM) or PBS solution. After EECS, the samples were rinsed thoroughly with PBS and stained with Calcein AM (Life Technologies, Grand Island, NY, USA). Three samples in each condition were used and in each sample five spots were randomly picked and the number of attached live cells were counted and used for the analysis (i.e., a total of 15 images). The surface area we investigated was 6 mm × 6 mm which was the size of thin film nitinol used in the cylindrical set-up and examined under the 1500× magnification.

Besides, LIVE/DEAD^®^ cell viability assay (Life Technologies, Grand Island, NY, USA) was done to determine the live attached cells using fluorescent microscopy (Olympus, Center Valley, PA, USA). Only green signals representing the live cells were analyzed. Both morphology and proliferation of adhered cells were evaluated using SEM after sputtering a thin palladium layer. For SEM imaging, cells were fixed with 2.5% glutaraldehyde (Sigma-Aldrich, St Louis, MO, USA), dehydrated in a series of ethanol/DI water mixtures including 30, 50, 75, 90, and 100% ratios, then, subjected to drying with hexamethyldisilazane (HMDS) (Alfa Aesar, Tewksbury, MA, USA)/ethanol in 3:1, 1:1, and 1:3 volume ratios. The samples were then dried in the chemical hood overnight at room temperature.

### 2.6. Statistical Analysis

The general linear model (GLM) can be written as:

(8) Y=XB+U

where, *Y* is a matrix with response measurements (i.e., attached number of cells), *X* is our design matrix (i.e., time duration, voltage, suspension solution, and substrate type), *B* is a matrix of the linear coefficient parameters to be estimated and *U* is the error matrix and assumed to be uncorrelated and follow a multivariate normal distribution. ANOVA then studies the significance of each predictor variable and *R^2^* statistic estimated the percentage of variation explained by the model.

Standard statistical analysis was conducted using Minitab 16 (Minitab Inc., State College, PA, USA) through calculation of the average factor effect (main effect), analysis of variance (ANOVA) using a general linear model, and testing the significance of factors’ influence. Using a main effects plot in Minitab examined the differences between level means for one or more factors.

## 3. Results and Discussion

### 3.1. Electrostatic Endothelial Cell Seeding in Parallel Capacitor-Like Set-Up

Since there were two four-level control factors (i.e., time and voltage) and two two-level control factors (i.e., CCM and substrate), L-16 array was selected without considering the interactions. Experimental plan can be found in Table 2, each row of this table shows an experiment with different combination of the parameter levels. The results of induced surface electrostatic charge in each of Taguchi designed experiments have been calculated based on the Equations (1)–(3).

**Figure 5:**
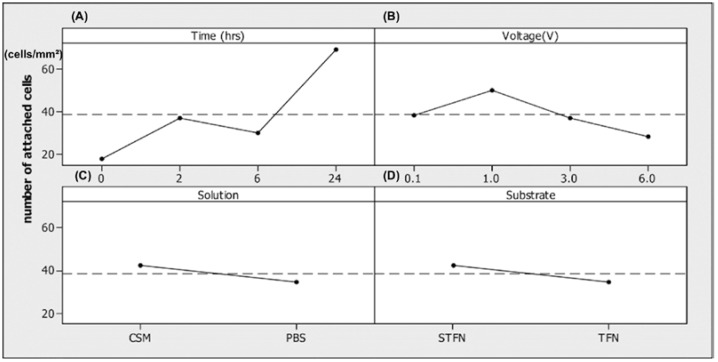
Diagram of the average effect: (**A**) time duration, (**B**) voltage, (**C**) cell suspension solution, and (**D**) substrate on the number of attached cells per mm^2^. The dashed line represents the overall mean of all the levels. The interaction time between cells and surface in total was 30 min and the incubation time after the stimulus was 0, 2, 6, and 24 h.

The results of the fluorescent imaging in each experimental condition described in Table 2 and the effect of changing the EECS parameters (i.e., voltage, time, substrate chemistry, and the suspension solution) on the average number of attached cells per mm^2^ were statistically analyzed. The results of the average number of attached cells per mm^2^ are shown in Figure 5 while the changing levels of each parameter. When the main effects plot line is horizontal, then there is no main effect for each factor level. On the other hand, the slope of the line represents the magnitude of the main effects. The average number of attached cells per mm^2^ significantly increased over time (Figure 5A). The average number of attached cells per mm^2^ increased through increasing the voltage from 0.1 V up to 1 V and the value reduced by decreasing the voltage from 1 V to 6 V; therefore, the average number of attached cells per mm^2^ was the highest in 1 V (Figure 5B). The result of the average effect of the CSM on the number of attached cells shows that this value was slightly higher in cell culture medium (~42) compared to it in the PBS (~35) (Figure 5C). Ultimately, the average number of attached cells per mm^2^ slightly increased on STFN (~43) compared to untreated TFN (~35) (Figure 5D). Figure 5 also identified two dominant factors (i.e., time duration and voltage) since these two plots showed relatively higher slopes compared to the variables including suspension solution and substrate type.

Analysis of variance (ANOVA) technique was performed to identify the significant factors that affect the number of attached cells. Thus, time and voltage with F-ratio of 42.67 (*p*-value < 0.001) and 7.12 (*p*-value = 0.016) were respectively identified as the two dominant factors. While solution and substrate parameters had F-ratio of 5.42 (*p*-value = 0.053) and 5.75 (*p*-value = 0.048), respectively. The adjusted R-square was 91.05%, suggesting a high correlation between the number of attached cells and predictor variables including time duration, voltage, cell suspension solution, and substrate type.

**Figure 6:**
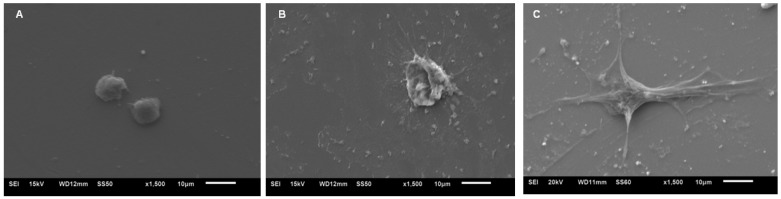
Representative SEM images of attached endothelial cells on STFN substrate in the cell culture medium solution after 2 h under (**A**) 0 V (Control), (**B**) 0.1 V, and (**C**) 1 V. The interaction time between cells and surface in total was 30 min and the incubation time after the stimulus was 0, 2, 6, and 24 h.

SEM images showed endothelial cells attached on STFN substrate after 2 h incubation in the cell medium exhibiting a few nanospikes emerging on the rounded-up cell bodies but upon applying 0.1 V, the number and length of these nanospikes significantly increased while under 1 V with the same condition, these cells become noticeably flat on the surface (Figure 6).

The ideal time for endothelialization could be at maximum a few hours in the catheter prior to the endovascular procedure. By increasing the incubation time from 2 h to 6 h, the cell morphology presumably became more flat and the nanospikes emerging on the surface remarkably lengthened. Applying 3 V caused cells to become more flat but cell membrane pitting significantly occurred indicating cell damage. By increasing the voltage up to 6 V, the level of disintegration and pitting in cell membrane significantly increases. Similar to STFN substrate, the cell membrane damage (i.e., cell membrane disintegration and pitting) under 6 V has also been observed in UTFN substrate in both PBS and cell culture medium while no cell membrane damage was observed in UTFN substrate under 3 V.

### 3.2. Electrostatic Endothelial Cell Seeding in Intravascular Catheter

Table 3 represents the value of applied electrical voltage, induced electrical field, and the induced electrostatic charge on the STFN substrate placed in the cylindrical capacitor setup of the intravascular catheter. Induced electrostatic charge and electric field was noticeably higher in the parallel capacitor set-up compared to the cylindrical capacitor setup of the intravascular catheter, for instance, under 1 V voltage, the induced electric field in parallel capacitor set-up and intravascular catheter set-up was 500 V/m, 1.022 V/m, respectively.

**Figure 7:**
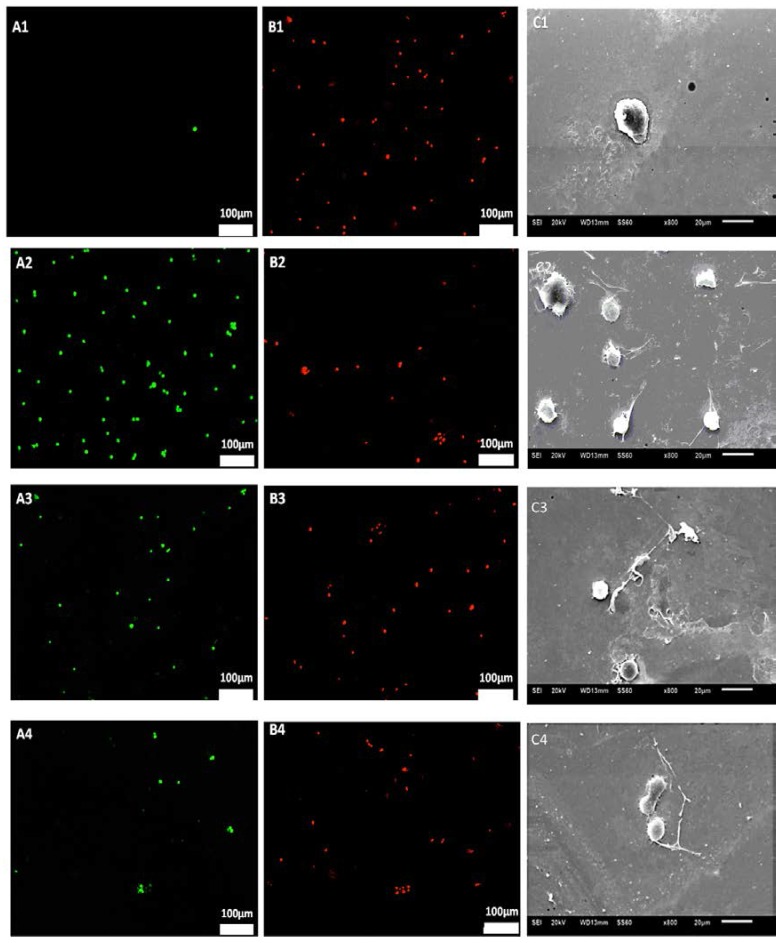
Live/Dead^®^ cell viability results of endothelial cell adherence on the STFN substrates after 2 h exposure: (**A1**,**B1**) 15 V, (**A2**,**B2**) 5 V, (**A3**,**B3**) 1 V and (**A4**,**B4**) 0 V (control UTFN) and SEM images of endothelial cells on the STFN substrates: (**C1**) 15 V, (**C2**) 5 V, (**C3**) 0.5 V and (**C4**) 0 V (control UTFN). The highest number of attached cells were observed in 5 V Condition (as seen in (**C3**)). The interaction time between cells and surface in total was 30 min and the incubation time after the stimulus was 0, 2, 6, and 24 h.

**Figure 8:**
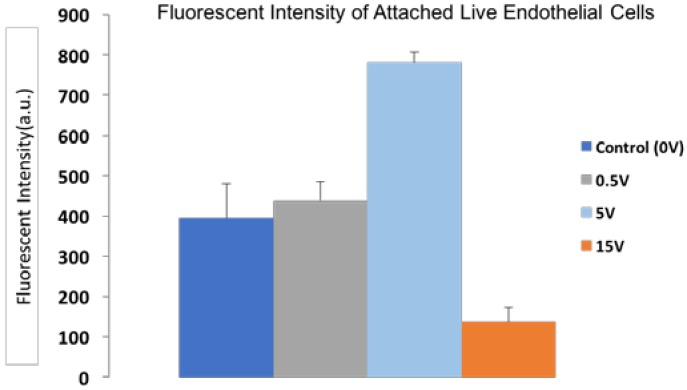
Fluorescent intensity of attached live Calcein AM stained endothelial cells under applying 0, 0.5, 5, and 15 V conditions.

Figure 7 shows the Live/Dead assay results and the SEM images of attached cells on STFN substrate placed in intravascular catheter setup when 1, 5, and 15 V were applied for 2 h. Figure 8 demonstrated the fluorescent intensity of live attached cells under 1, 5, and 15 V. The number of attached live EC increased under 1 V and 5 V while the number of live cells under 15 V significantly decreased compared to the control condition (0 V). The highest number of attached cells was observed in the 5 V condition. The morphology of cells under the condition of applying electrical voltage was similar to the control sample but under 5 V, multiple podia formed on the surface of the cell showing the advancement of cell maturation in this condition.

The cell is the basic structural unit of all living organisms. One of the important features of biological cells is having electrical nature due to the exchange of electrically charged ions across the cell membrane and showing the electrical potential of its interior relative to the exterior. Cell membranes consist of phospholipid bilayer structure and ion channels (i.e., K and Cl ion channels) and proteins are embedded across this phospholipid bilayer. Phospholipid bilayer in the cell membrane is an insulator layer that acts as the dielectric separating two electrically conductive regions of the cytoplasm and the extracellular fluid. Therefore, the cell can be modeled by a capacitor. Electric fields exist in many biological systems and influence cell signaling as well as many cellular behaviors and biological events. Therefore, electrophysiological features of cells vary with the surrounding physiological environment. At rest, most cells have typically a potential around −40 to −80 mV indicating that they are dominated by K or Cl permeability. In a study by Jongsma et al., the membrane potential, capacitance, and input resistance of isolated human vascular endothelial cells were measured as −16.3 ± 12.7 mV, 53.9 ± 5.5 pF, and 2.3 ± 1.3 GΩ [20].

Based on the electrophysiological features of cells, the cells can be assumed as charged particles and EECS can be modeled as electrophoretic deposition (EPD) [21]. EPD is a process that colloidal charged particles suspended in a solution and they migrate under the influence of an electric field generated between two electrodes. Thus, they are deposited onto an electrode based on its electrostatic charge. In the EECS set-up that we used in this study, the TFN plays as the positive electrode and the endothelial cells that are suspended in the PBS or cell culture medium (CCM) solution play as the electrostatic negatively charged particles. Avgustinik et al., described the factors affecting the amount of deposited particles during the EPD process in cylindrical, coaxial electrodes. These factors include the length (*l*) and radius (*a*) of the deposition electrode, the radius of the coaxial counter electrode (*b* > *a*), the particle mass concentration in the suspension (*C*), permittivity (*ε*), zeta potential (*ξ*), viscosity of the suspension (*η*), electric field strength (*E*), and the deposition time (*t*) [22,23,24].

In this study, the effects of four factors (i.e., voltage, substrate, solution, and incubation time) in the parallel EECS setup were studied using Taguchi Design of Experiment (DOE) method. Since parallel capacitor setup is simpler to perform the EECS experiments compared to the cylindrical capacitor intravascular setup, it was first used to study the effects of EECS parameters [25]. Design of the experiment method allows evaluating multiple factors simultaneously and finding the optimal condition for accelerating and improving the EECS on the various TFN substrates.

Both PBS and cell culture medium (CCM) solutions were used for testing the electrostatic cell seeding effectiveness of TFN materials. While dielectric constants of these two solutions are not significantly different, the main difference between them is the presence of proteins in the cell medium. Our results demonstrated that the average number of attached cells was higher when the cell medium solution was used. Proteins in biological serum effectively play a role in the cell adhesion process such that proteins and biomolecules first adsorb on the surface of materials and then cells attach on these pre-adsorbed proteins. Proteins are large biomolecules and contain multiple functional groups with electrostatic charge; therefore, the electrostatic charge of the substrate can affect the type and the arrangement of the deposited proteins and subsequently impact their function, ligand binding, and cell signaling. The estimation of the average effect of voltage showed that increasing voltage up to an optimal range can enhance cell attachment but increasing it beyond this point decreased the number of attached cells. 

The applied voltage also affects both the induced electric field and substrate electrostatic charges. McCaig et al., showed electric fields in the range of 75–100 mV·mm^−1^ with voltage of 1.5–2.0 mV enhanced the elongation and migration of endothelial cells [26], which would be beneficial for better endothelialization. If the applied voltage across the capacitor becomes too large, the dielectric breaks down and results in a short circuit. In addition, high voltage and a high electric field can damage the cell membrane. For instance, the number of attached cells was decreased above 15 V applied voltage in the cylindrical capacitor test setup compared to the control (0 V applied voltage) or the voltage below 15 V.

The incubation time did not significantly influence the number of endothelial cells adhered, however, qualitative evaluation of the adhered cells by SEM images showed that the cells became flatter and lengthened on all TFN materials. The average number of attached cells was slightly higher on the STFN substrate compared to the UTFN. Even though endothelial dysfunction could play an important role in the cell seeding process and affect the properties of the thin film nitinol, future studies designed to understand the influence of vascular tone, permeability, and platelet properties could effectively address their effect.

## 4. Conclusions

One of the major failures in the current small-diameter vascular grafts may be caused by a lack of sufficient endothelialization and subsequent thrombosis occurrence. Recent advancement in thin film nitinol (TFN) endovascular grafts suggested this material as an alternative for existing synthetic polymer graft materials. TFN shows several superior properties such as superelasticity, shape memory effect, biocompatibility, and ultra low-profile feature. In addition to these unique features, electrostatic endothelial cell seeding (EECS) of the collapsed device prior to the endovascular procedure was proposed in order to seed endothelial cells on the surface of TFN to enhance the biocompatibility.

The condition of 5 V voltage with an induced electrostatic charge of 0.68 × 10^−6^ µC and induced electric field of 5.11 V/mm (i.e., a much lower amount compared to the parallel plate capacitor setup) showed the highest endothelial cell attachment in the cylindrical setup with an intravascular catheter. Therefore, the collapsed STFN endograft within the 6 Fr size intravascular catheter can be successfully endothelialized under 5 V for 2 h. In future work, the phenotypic features of attached endothelial cells and the in vivo studies of implanted electrostatic endothelial cell seeded TFN will be evaluated in detail to understand the mechanism for utilization of electrostatic endothelial cell seeding in the endovascular device arena.

## Figures and Tables

**Table 1:** Electrostatic cell seeding (EECS) parameters and their levels.

Level	Time (h)	Voltage (V)	Suspension Solution	Substrate
1	0	0.1	PBS	UTFN
2	2	1	Cell Culture Medium (CCM)	STFN
3	6	3	–	–
4	24	6	–	–

**Table 2:** Experimental plan using L-16 orthogonal array along with induced electrostatic charge and electrical field in the designed experiments.

Experiment Number	Time (h)	Voltage (V)	Solution	Substrate	Electrostatic Charge (µC)	Electrical Field (V/m)
1	0	0.1	CCM	UTFN	3.18 × 10^−7^	50
2	0	1	CCM	UTFN	3.18 × 10^−6^	500
3	0	3	PBS	STFN	9.54 × 10^−6^	1500
4	0	6	PBS	STFN	1.9 × 10^−5^	3000
5	2	0.1	CCM	STFN	3.18 × 10^−7^	50
6	2	1	CCM	STFN	3.18 × 10^−6^	500
7	2	3	PBS	UTFN	9.54 × 10^−6^	1500
8	2	6	PBS	UTFN	1.9 × 10^−5^	3000
9	6	0.1	PBS	UTFN	3.18 × 10^−7^	50
10	6	1	PBS	UTFN	3.18 × 10^−6^	500
11	6	3	CCM	STFN	9.54 × 10^−6^	1500
12	6	6	CCM	STFN	1.9 × 10^−5^	3000
13	24	0.1	PBS	STFN	3.18 × 10^−7^	50
14	24	1	PBS	STFN	3.18 × 10^−6^	500
15	24	3	CCM	UTFN	9.54 × 10^−6^	1500
16	24	6	CCM	UTFN	1.9 × 10^−5^	3000

**Table 3:** Electrostatic cell seeding (EECS) experiments in intravascular catheter.

Experiment Number	Voltage (V)	Electrostatic Charge (µC)	Electrical Field (V/mm)
1	1	0.136 × 10^−6^	1.022
2	5	0.68 × 10^−6^	5.11
3	15	2.04 × 10^−6^	15.33
